# Perceptions of Doctors in Saudi Arabia Toward Virtual Reality and Augmented Reality Applications in Healthcare

**DOI:** 10.7759/cureus.42648

**Published:** 2023-07-29

**Authors:** Wareef A Alhumaidi, Noura N Alqurashi, Razan D Alnumani, Ebtehal S Althagafi, Fatimah R Bajunaid, Ghaliah O Alnefaie

**Affiliations:** 1 Department of Medicine and Surgery, Taif University, Taif, SAU; 2 Department of Pathology, Taif University, Taif, SAU

**Keywords:** physicians’ perception, medicine, healthcare, virtual reality, technology, augmented reality, artificial intelligence

## Abstract

Background

Several studies suggested that artificial intelligence (AI), including virtual reality (VR) and augmented reality (AR), may help improve visualization, diagnostic, and therapeutic abilities and reduce medical and surgical errors. These technologies have been revolutionary in Saudi Arabia. We aimed to elucidate physicians' perceptions toward these technologies.

Methodology

We carried out a cross-sectional electronic questionnaire-based study in November 2021. The study targeted doctors of different medical and surgical specialties in the western region of Saudi Arabia.

Results

In our study, 53.2% of the participants were 25-30 years old. Most participants were residents (53.6%) with career experiences <5 years. Only 32.3% had a good familiarity with AR and VR technologies. However, 64.5% agreed that AR and VR technologies had practical applications in the medical field. Moreover, 35% agreed that the diagnostic and therapeutic ability was superior to the clinical experience of a human doctor. About 41.4% agreed they would always use AR and VR technologies for future medical decisions.

Conclusion

Doctors are open to using AR and VR technologies in healthcare. Although most people are unfamiliar with these technologies, most agree that they positively impact healthcare.

## Introduction

Artificial intelligence (AI), augmented reality (AR), virtual reality (VR), and machine learning are permeating all aspects of healthcare [1]. The use of AR in medicine officially began [2] with the addition of computed tomography images to a monoscopic operating microscope for neurosurgery in the 1980s. A head-mounted display with video see-through was created for enhanced ultrasound imaging in the early 1990s [3]. VR and AR are digital technologies that allow automation and can be employed in situations where repetitive actions must be carried out frequently [2]. Both aim to provide people with audio and visual experiences by simulating the world [1,2].

Recent technological developments have improved the portability, realism, and real-time navigation of VR and AR systems and expanded the sensory range by adding sensory and occasionally olfactory aspects [1]. VR is a technology that uses a portable screen in the form of a VR headset to immerse people in synthetic three-dimensional environments. It simulates a situation rather than altering reality. It has been applied to video games, design planning, and roller coaster simulations [4]. Anatomical models, preoperative photographs, and cadavers for planning operations may be a part of the medical field's VR application. It was created to make ORs safer for patients during surgical training and practice. In preoperative planning, VR can be used with AI algorithms to help surgeons map operations realistically [4].

On the other hand, the closely linked AR uses VR components to overlay them in the real world using live videos presented on an electronic device [3]. AR has largely improved robotic surgery because there are no involved simulations; instead, natural occurrences are changed in real real-time to enhance patient safety; AR is currently used to overlay important anatomical features during live surgeries [1]. Telemanipulator devices can be combined with AR to improve OR visibility [5]. VR and AR can be utilized in surgical procedure training to allow trainees to conduct procedures on a virtual patient or to see information about the patient superimposed on reality [6-8]. Several studies have suggested that VR may improve surgical skills and reduce errors [8]. This study aimed to assess doctors' perceptions in Saudi Arabia toward the implications of AR and VR technologies in medicine.

## Materials and methods

Study design and settings

This cross-sectional electronic questionnaire-based study was carried out in November 2021. This study targeted doctors of different medical and surgical specialties in the western region of Saudi Arabia. The research team collected data using an online questionnaire conducted via social media websites.

Sample population

The total sample size was 220 people. The questionnaire links were sent to each participant. Each specialist received the same connection to ensure each participant was represented correctly.

Questionnaire instrument

The questionnaire's content was identical across all links. In addition to the link to social media, a brief introduction was provided regarding the study's objectives and goals. We informed the participants that the participation was voluntary and anonymous and that all information would be kept strictly confidential. The participants then had to confirm their involvement in the study by selecting either "agree" to continue the research or "disagree" with the participant's anonymous identities. The study's primary inclusion criteria were (1) doctors who work in different specialties of medicine in Saudi Arabia, (2) those who confirm their agreement to answer the questionnaire, and (3) those who completed the questionnaire. The study's primary exclusion criteria were (1) undergraduate medical students, (2) healthcare workers other than doctors (pharmacists, physiotherapists, nurses, laboratory specialists), (2) those who did not confirm their agreement to answer the questionnaire, and (3) those who did not complete the questionnaire. Using the Google questionnaire, we designed an electronic questionnaire consisting of three parts. The first part was the participants’ personal information, including the participants’ place of residence, gender, age, years of experience as a carrier, professional title, and specialty. The second part was about the participant's basic understanding of AR and VR, including whether the participant had sufficient information about AR and VR technologies. The third part was about the participant's attitudes to AR and VR, including whether they thought the AR anthologies would replace doctors.

Face validity check and piloting phase

The questionnaire was piloted on a group of students (n = 20) to analyze its validity and ensure a proper interpretation of the questions. The results of the pilot questions appeared satisfactory and valid.

Statistical analysis

The results of the questionnaires were displayed in Excel version 16.16.23 (Microsoft, Washington, USA), while the data were analyzed statistically using SPSS Statistics version 26 (IBM Corp. Released 2019. IBM SPSS Statistics for Windows, Version 26.0. Armonk, NY: IBM Corp.). The questionnaire used in this study included questions on Saudi doctors' perceptions of using logics in medicine. The questionnaire items were derived from previous studies in [9] and other countries [10,11]. A standardized methodology was used to validate the questionnaire, which included focus group discussions, expert evaluation, pilot study, and reliability and validity assessments. A pilot study with 20 participants was carried out. The results were used for reliability and validity analyses. The content and face validity of the questionnaire were evaluated using expert evaluations and focused-group discussions. An exploratory factor analysis was used to test the construct validity of the questionnaire. Items with correlation coefficients higher than 0.7 were eliminated. The reliability of the questionnaire was assessed. The Cronbach's alpha value was estimated to be 0.9. A Cronbach's coefficient above 0.7 indicates that the questionnaire is internally consistent [12].

Ethical approval

Taif University's ethics committee approved this study and approved the research proposal. The National Committee for Bioethics accredits the committee with No. HAO-02-T-105.

## Results

About 53.2% of the participants were 25-30 years old, 54.1% were males, and 47.7% had a career experience <5 years. Among them, 53.6% were residents (Table 1).

**Table 1:** Distribution of studied participants according to their demographic and work characteristics GP: general practitioner

Variable	N	%
Age (years)		
25-30	117	53.20%
30-35	27	12.30%
35-40	23	10.50%
40-45	23	10.5%
45-50	11	5%
>50	19	8.60%
Gender		
Female	101	45.9%
Male	119	54.10%
Career experience (years)		
<5	115	47.70%
05-Oct	14	6.4%
15-Oct	27	12.30%
15-20	22	10%
20-25	15	6.80%
>25	37	16.80%
Position		
Consultant	47	21.40%
Fellow	11	5%
GP	4	1.80%
Intern	6	2.70%
Resident	118	53.60%
Specialist	34	15.50%

Participants were asked about their familiarity with AR and VR in the perception survey. Only 32.3% of the participants agreed they were familiar with AR and VR technologies. However, 64.5% agreed that AR and VR technologies have useful applications in the medical field. Among them, 42.7% agreed that the visualization ability of the AR and VR technologies was superior to that of a human doctor with clinical experience. Approximately 37% (37.3%) and 35% agreed that AR and VR technologies' diagnostic and therapeutic abilities are superior to human doctors' clinical experience, respectively. Only 24.6% agreed that AR and VR technologies could replace their jobs, and 41.4% agreed that they would always use AR and VR technologies for medical decisions in the future. More than half (58.1% and 60.9%) agreed that AR and VR technologies could increase the speed of processes in healthcare and reduce medical errors, respectively. Approximately 53% (53.2%) agreed that AR and VR technologies could reduce burnout among doctors, and 35.4% agreed that these technologies have no physical limitations (Table 2).

**Table 2:** Distribution of studied participants according to their response to knowledge and attitude items related to AR and VR technology AR: augmented reality, VR: virtual reality

Variable	Strongly agree	Agree	Neutral	Disagree	Strongly disagree
Do you agree that you have good familiarity with AR and VR technology?	23 (10.5)	48 (21.8)	77 (35)	47 (21.4)	25 (11.4)
Do you agree that AR and VR technology have useful applications in the medical field?	72 (32.7)	70 (31.8)	57 (25.9)	10 (4.5)	11 (5)
Do you agree that the visualization ability of AR and VR technology is superior to the clinical experience of human doctors?	37 (16.8)	57 (25.9)	66 (30)	41 (18.6)	19 (8.6)
Do you agree that the diagnostic ability of AR and VR technology is superior to the clinical experience of human doctors?	25 (11.4)	50 (22.7)	66 (30)	59 (26.8)	20 (9.1)
Do you agree that the therapeutic ability of AR and VR technology is superior to the clinical experience of human doctors?	23 (10.5)	54 (24.5)	81 (36.8)	44 (20)	18 (8.2)
Do you agree that AR and VR technology could replace your job?	12 (5.5)	42 (19.1)	59 (26.8)	60 (27.3)	47 (21.4)
Do you agree that you would always use AR and VR technology when making medical decisions in the future?	20 (9.1)	71 (32.3)	90 (40.9)	29 (13.2)	10 (4.5)
Do you agree that the usage of AR and VR technology can speed up processes in healthcare?	41 (18.6)	87 (39.5)	71 (32.3)	14 (6.4)	7 (3.2)
Do you agree that the usage of AR and VR technology can reduce medical errors?	42 (19.1)	92 (41.8)	61 (27.7)	17 (7.7)	8 (3.6)
Do you agree that the usage of AR and VR technology can reduce burnout among doctors?	28 (12.7)	89 (40.5)	79 (35.9)	18 (8.2)	6 (2.7)
Do you that AR and VR technology has no physical limitations?	21 (9.5)	57 (25.9)	75 (34.1)	35 (15.9)	32 (14.5)

Participants with sufficient general information about AR and VR technologies and who did not know about AR and VR applications in the medical field had a significantly higher percentage of those with positive responses toward AR and VR technologies (p <0.05). In comparison, a nonsignificant relationship was observed between the participants' demographic and work characteristics (p >0.05) (Table 3).

**Table 3:** Relationship between participants' attitude toward AR and VR technology and their demographic and work characteristics, having enough information about AR and VR technology in general and knowledge about the applications of AR and VR in the medical field AR: augmented reality, VR: virtual reality, GP: general practitioner

Variable	Attitude		Chi-square test	p-value
	Negative N (%)	Positive N (%)		
Age (years)				
25-30	46 (46)	71 (59.2)	9.45	0.092
30-35	15 (15)	12 (10)		
35-40	10 (10)	13 (10.8)		
40-45	40 (40)	12 (10)		
45-50	4 (4)	7 (5.8)		
>50	14 (14)	5 (4.2)		
Gender				
Female	45 (45)	56 (46.7)	0.61	0.805
Male	55 (55)	64 (53.3)		
Career experience (years)				
<5	46 (46)	59 (49.2)	2.86	0.721
05-Oct	15 (15)	22 (18.3)		
Oct-15	12 (12)	15 (12.5)		
15-20	10 (10)	12 (10)		
20-25	8 (8)	7 (5.8)		
>25	9 (9)	5 (4.2)		
Position				
Consultant	21 (21)	26 (21.7)	5.69	0.337
Fellow	7 (7)	4 (3.3)		
GP	1 (1)	3 (2.5)		
Intern	2 (2)	4 (3.3)		
Resident	49 (49)	69 (57.5)		
Specialist	20 (20)	14 (11.7)		
Do you have enough information about AR and VR technology in general?				
No	66 (66)	58 (48.3)	6.92	0.009
Yes	34 (34)	62 (51.7)		
Do you know about AR and VR applications in the medical field?				
No	71 (71)	63 (52.5)	7.84	0.005
Yes	29 (29)	57 (47.5)		

**Figure 1:**
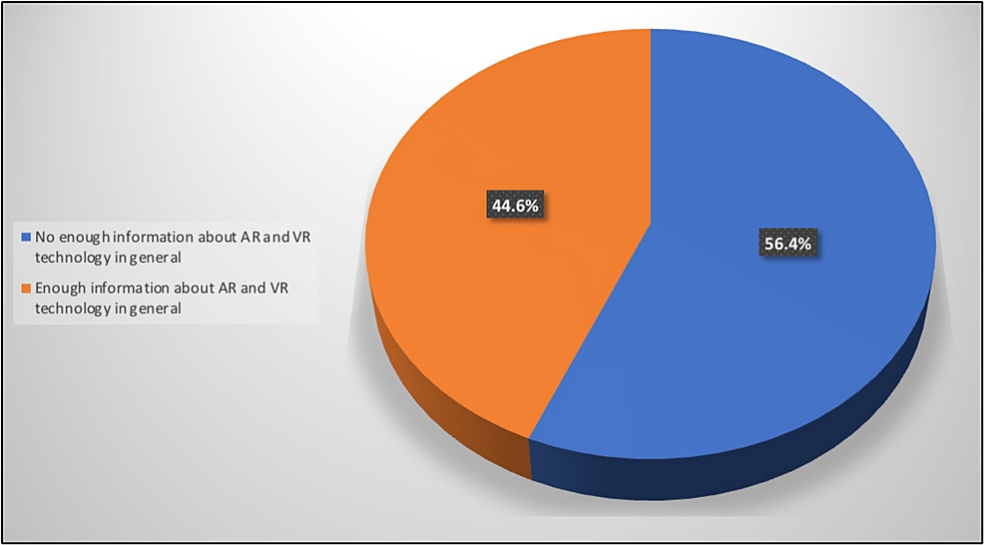
Percentage distribution of studied participants according to having enough information about AR and VR technology in general AR: augmented reality, VR: virtual reality

**Figure 2:**
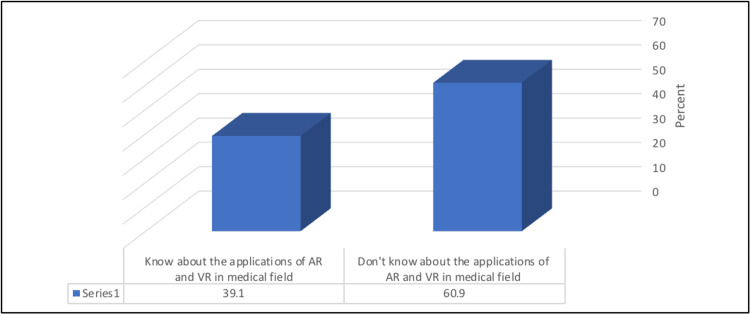
Percentage distribution of studied participants according to their knowledge about the applications of AR and VR in the medical field AR: augmented reality, VR: virtual reality

Approximately 44% (44.6%) of the participants had sufficient general information about AR and VR technologies. Moreover, 39.1% reported knowing about AR and VR applications in the medical field (Figures 1-2).

**Figure 3:**
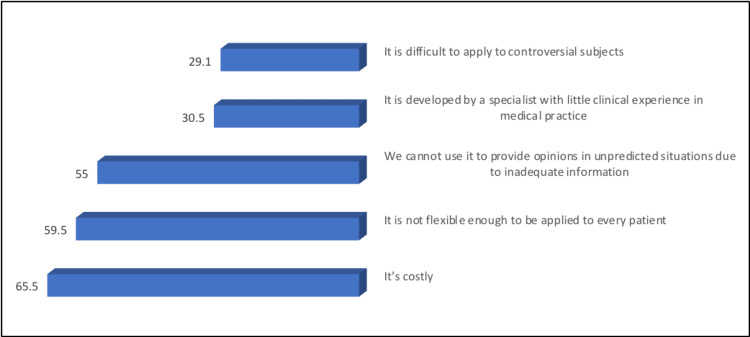
Percentage distribution of studied participants according to their concerns about the application of AR and VR technologies in medicine

The most common concerns of the participants regarding the application of AR and VR technologies in medicine were the cost (65.5%) and non-flexibility for application to every patient (59.5%). They cannot be used to provide opinions in unpredictable situations owing to inadequate information (55%) (Figure 3).

**Figure 4:**
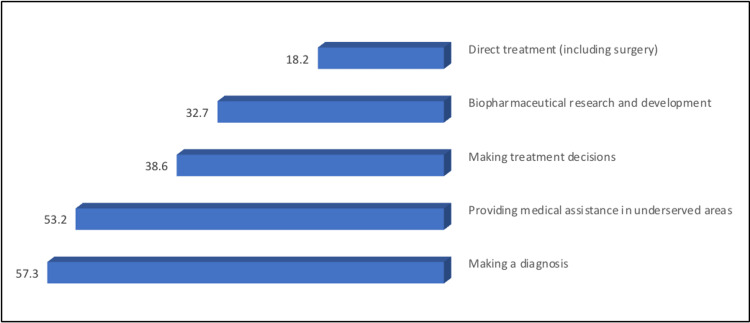
The percentage distribution of study participants according to their responses about the medical applications of AR and VR technologies will be most helpful

The most commonly reported medical applications, the AR and VR technologies, will be most helpful, according to the participants' opinions, in diagnosis (57.3%) and medical assistance in underserved areas (53.2%) (Figure 4).

**Figure 5:**
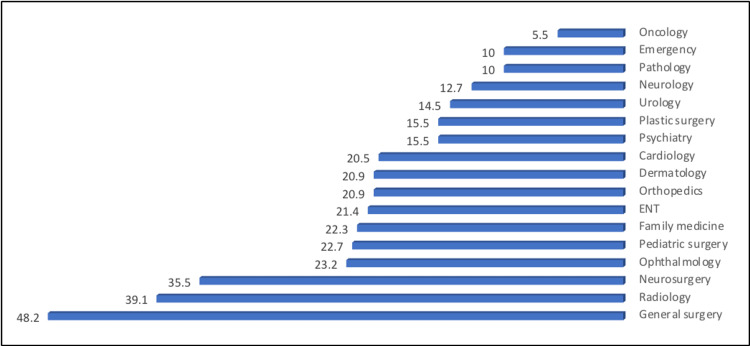
The percentage distribution of study participants according to their responses about the medical field of AR and VR technologies will be most helpful

The most common medical fields were general surgery (48.2%), radiology (39.1%), and neurosurgery (35.5%) (Figure 5).

## Discussion

Our study showed that approximately a quarter of the participants were familiar with AR and VR technologies and that most agreed on their usefulness in medical technologies. In addition, similar trends were observed in a Swiss study where Staartjes et al. surveyed that most respondents agreed with the practicality and deployment of machines (AR and VR) in their medical practices [13]. Furthermore, in another study in the United Kingdom, more than half of the individuals agreed with the significance of AR in the medical field [14].

These technologies are superior regarding many physicians' diagnoses and clinical experience. A study in Germany showed that more than half of the students felt more integrated, which would improve the radiological field. Similarly, Buchanan showed that students trained with VR exhibited better and faster learning experiences, almost at the same level as traditional medical practice trainees [15].

In our study, some individuals agreed that VR and AR technologies could accelerate healthcare. Similar results were obtained in a survey by Hui et al., where the subjects exhibited significant improvements in their cognitive function and processing speed [16]. Another study in Korea showed that adult patients with mild mental disabilities improved their cognitive function using VR technology. These results support our participants' perceptions of enhancing the patient's recovery and healthcare processes [17].

According to our findings, a minority of respondents believed these technologies are useful in medical decisions. Approximately half of the individuals were confident they could help reduce medical errors. Similar studies demonstrated that practitioners who practiced surgery with VR and AR technologies before the surgery performed better with fewer errors than their fellow residents [18]. Similar findings were obtained at Shiraz University of Medical Sciences, when trainees used the VR technology, with higher accuracy and fewer errors than in the conventional training [19]. A few respondents believed that these technologies could replace their jobs. Numerous studies have revealed the effectiveness of AR and VR in reducing errors and improving procedural safety, skill acquisition, and senior supervision time. However, they cannot eliminate human presence in such circumstances [20]. AR and VR technologies and programs, such as virtual standardized patients, help physicians make better differential diagnoses and clinical decisions [21]. This will improve the practitioners' situational awareness and increase the participants' trust.

Approximately half of the individuals believed that they could relieve the burnout of practitioners. A study at Mount Sinai demonstrated that using VR technologies on their residents for two months can significantly reduce emotional exhaustion (P = 0.027) and residents' burnout rate [22]. A minority of respondents in our study indicated that VR and AR technologies have no physical limitations. However, the rules of these technologies have been confirmed. AR and VR require continuous data regulation, AI, and algorithms for practical statistical and interpretive data analyses [23]. Our study indicated that most individuals believed this has a higher cost; however, as a contrasting opinion, Lan et al. stated that VR could be cost-effective in surgery and clinical medicine. An increase in the number of trainees is associated with increased resource availability. VR and AR can significantly reduce the need for other resources and present themselves as cost-effective approaches to training and practicing [24].

## Conclusions

Doctors are open to the use of AR and VR technologies in healthcare. Although most people are unfamiliar with these technologies, the majority agree that they positively impact healthcare. They believe that these technologies will help reduce medical errors and practitioner burnout. However, current barriers must be resolved to increase the awareness and use of these technologies for some healthcare professionals in the future.

## Appendices

**Table 4:** AR-VR questionnaire AR: augmented reality, VR: virtual reality

Question	Type	Options
Do you agree to answer this survey?	Yes/No	Yes, No.
Are you a doctor in Saudi Arabia?	Yes/No	Yes, No.
Your region?	Multiple choice	Western, Eastern, Southern, Northern, Central.
Your gender?	Multiple choice	Male, Female.
How old are you?	Multiple choice	25-30, 30-35, 35-40, 40-45, 45-50, >50.
Years of experience in your career?	Multiple choice	<5, 5-10, 10-15, 15-20, 20-25, >25.
Are you?	Multiple choice	Resident, specialist, fellow, consultant.
What is your specialty?	Multiple choice	General surgery, neurosurgery, pediatric surgery, ENT, ophthalmology, radiology, family medicine, neurology, dermatology, cardiac surgery, orthopedic, urology, and plastic surgery.
Do you have enough information about AR and VR technology in general?	Yes/No	Yes, No.
Do you know about the applications of AR and VR in the medical field?	Yes/No	Yes, No
Do you agree that AR and VR technologies can be used in the medical field?	Likert scale	Strongly disagree, disagree, neutral, agree, strongly agree.
Do you agree that the visualization ability of AR and VR technology is superior to the clinical experience of human doctors?	Likert scale	Strongly disagree, disagree, neutral, agree, strongly agree.
Do you agree that the diagnostic capacity of AR and VR technologies is superior to that of a human doctor's clinical experience?	Likert scale	Strongly disagree, disagree, neutral, agree, strongly agree.
Do you agree that the therapeutic capacity of AR and VR technologies is superior to that of a human doctor's clinical experience?	Likert scale	Strongly disagree, disagree, neutral, agree, strongly agree.
Do you agree that AR and VR technology could replace your job?	Likert scale	Strongly disagree, disagree, neutral, agree, strongly agree.
Do you agree that AR and VR technology have the potential to replace your job?	Likert scale	Strongly disagree, disagree, neutral, agree, strongly agree.
Do you agree that using AR and VR technologies can help to speed up processes in healthcare?	Likert scale	Strongly disagree, disagree, neutral, agree, strongly agree.
Do you agree that the use of AR and VR technology can help to reduce medical errors?	Likert scale	Strongly disagree, disagree, neutral, agree, strongly agree.
Do you agree that using AR and VR technologies might help doctors minimize burnout?	Likert scale	Strongly disagree, disagree, neutral, agree, strongly agree.
Do you agree that AR and VR technology has no physical constraints?	Likert scale	Strongly disagree, disagree, neutral, agree, strongly agree.
What concerns do you have regarding the use of AR and VR technologies in medicine? (You may select more than one option.)	Multiple choice	We cannot use it to deliver opinions in unexpected scenarios due to a lack of information, and it is not flexible enough to be applied to every patient. It is difficult to apply to contentious issues, it is pricey, and it was created by a professional with no practical expertise in medical practice.
Which of the following do you believe AR/VR technology will be most useful? (You may select more than one option.)	Multiple choice	Making a diagnosis, making treatment decisions, direct treatment (including surgery), biopharmaceutical research and development, providing medical assistance in underserved areas.
In which field of medicine do you think AR and VR technology will be most useful?	Multiple choice	General surgery, neurosurgery, pediatric surgery, ENT, ophthalmology, radiology, family medicine, neurology, dermatology, cardiac surgery, orthopedic, urology, plastic surgery.
